# Characterisation of myocardial structure and function in adult-onset growth hormone deficiency using cardiac magnetic resonance

**DOI:** 10.1007/s12020-016-1067-6

**Published:** 2016-08-17

**Authors:** Julia D.J. Thomas, Abhishek Dattani, Filip Zemrak, Thomas Burchell, Scott A. Akker, Mark Gurnell, Ashley B. Grossman, L. Ceri Davies, Márta Korbonits

**Affiliations:** 1 Department of Endocrinology, William Harvey Research Institute, Barts and the London School of Medicine and Dentistry, Queen Mary University of London, Charterhouse Square, London, EC1M 6BQ UK; 2 NIHR Cardiovascular Biomedical Research Unit, Barts Heart Centre, St Bartholomew’s Hospital, London, UK; 3 Department of Endocrinology, St Bartholomew’s Hospital, London, UK; 4 University of Cambridge, Metabolic Research Laboratories, Addenbrooke’s Hospital, Cambridge, UK; 5 Oxford Centre for Endocrinology, Diabetes and Metabolism, University of Oxford, Oxford, UK

## Abstract

Growth hormone (GH) can profoundly influence cardiac function. While GH excess causes well-defined cardiac pathology, fewer data are available regarding the more subtle cardiac changes seen in GH deficiency (GHD). This preliminary study uses cardiac magnetic resonance imaging (CMR) to assess myocardial structure and function in GHD. Ten adult-onset GHD patients underwent CMR, before and after 6 and 12 months of GH replacement. They were compared to 10 age-matched healthy controls and sex-matched healthy controls. Left ventricular (LV) mass index (LVMi) increased with 1 year of GH replacement (53.8 vs. 57.0 vs. 57.3 g/m^2^, analysis of variance *p* = 0.0229). Compared to controls, patients showed a trend towards reduced LVMi at baseline (51.4 vs*.* 60.0 g/m^2^, *p* = 0.0615); this difference was lost by 1 year of GH treatment (57.3 vs. 59.9 g/m^2^, *p* = 0.666). Significantly reduced aortic area was observed in GHD (13.2 vs. 19.0 cm^2^/m^2^, *p* = 0.001). This did not change with GH treatment. There were no differences in other LV parameters including end-diastolic volume index (EDVi), end-systolic volume index, stroke volume index (SVi), cardiac index and ejection fraction. There was a trend towards reduced baseline right ventricular (RV)SVi (44.1 vs. 49.1 ml/m^2^, *p* = 0.0793) and increased RVEDVi over 1 year (70.3 vs. 74.3 vs. 73.8 ml/m^2^, *p* = 0.062). Two patients demonstrated interstitial expansion, for example with fibrosis, and three myocardial ischaemia as assessed by late gadolinium enhancement and stress perfusion. The increased sensitivity of CMR to subtle cardiac changes demonstrates that adult-onset GHD patients have reduced aortic area and LVMi increases after 1 year of GH treatment. These early data should be studied in larger studies in the future.

## Introduction

Growth hormone deficiency (GHD) causes myocardial hypokinesis, with loss of the normal response to exercise [1–5]. Effects of GHD on cardiac mass have been most clearly demonstrated in childhood-onset GHD (CO-GHD) but the impact of adult-onset GHD (AO-GHD) remains controversial, with variable conclusions in different studies [6–10]. The difficulties defining the effect of AO-GHD on cardiac mass are due, in part, to limitations in traditional methods of assessment [11, 12]. GH replacement improves exercise tolerance, but it is unclear if this is due to changes in cardiac mass, function or circulating volume [3]. Indeed, there are several ways in which GH is thought to act on the heart with GH excess leading to a specific cardiomyopathy characterised by concentric ventricular hypertrophy and biventricular involvement [13]. If GH excess is left untreated, cardiac failure develops [1]. The aetiology of this change is thought to include both direct and indirect GH action, via the effect of insulin-like growth factor-1 (IGF-1) produced by the action of GH on the GH receptor. IGF-1 excess causes increased synthesis of myosin light chain-2 and troponin I, leading to cardiac cell growth [14]. IGF-1 is also known to increase intracellular calcium and increase calcium sensitivity of the cardiac myocytes leading to increased cardiac contractility [15]. The administration of recombinant IGF-1 in healthy subjects increases cardiac performance, thought to be due to direct cardiac action as well as a vasodilatory effect of IGF-1 via endothelial nitric oxide synthase (eNOS) [16]. Furthermore, GH has effects on salt and water handling via the kidney and GH excess increases circulating volume [17]. It is clear that the GH/IGF-1 system interacts with the cardiovascular system in multiple ways and the cardiac pathology seen in patients with GH excess and deficiency will arise due to a range of interactions.

Cardiac magnetic resonance (CMR) imaging is a reliable and reproducible technique for measuring myocardial volume, mass and function. The use of gadolinium contrast enables assessment of myocardial perfusion, scarring and focal fibrosis [18]. CMR has been used to study a range of cardiomyopathies, including hypertrophic cardiomyopathy, Marfan and Turner syndromes [19–21]. Currently, there is only one other CMR study assessing the impact of GH deficiency on the heart and this study confined itself to reporting the effects on the left ventricle (LV) [22]. The effects of GH deficiency on the right ventricle (RV) and aortic area (AA) have not previously been studied in these patients using CMR. Our study has sought to examine the LV, RV and AA in this patient group, to look for evidence of interstitial expansion, for example, with myocardial fibrosis as demonstrated by late gadolinium enhancement (LGE) and to use stress CMR and perfusion techniques to explore the presence of ischaemic heart disease, often suggested as a confounder in the assessment of GH-related cardiac disease.

## Subjects and Methods

### Patients

Ten adult patients with GHD about to commence GH replacement were recruited from two UK endocrine centres and were studied in the main study centre. GHD was confirmed by insulin tolerance or glucagon testing (peak GH < 3.0 µg/l). Cardiac function and morphology were assessed using CMR before initiation of the chosen therapy (CMR1) and at 6 (CMR2) and 12 months (CMR3) after treatment. Patients provided quality of life (QoL) data using the Adult Growth Hormone Deficiency Assessment, a disease-specific questionnaire, weight, height and blood pressure measurements and serum IGF-1 levels at each time point. Patients with known ischaemic heart disease or hypertension before the study were excluded. Multi-centre ethical approval was given by the local Research Ethics Committee (06/Q0401/53). This was an observational study and referring physicians determined patient treatment according to the local guidelines. All patients signed a consent form following verbal and written explanation of the study.

#### GHD group

Ten patients (two female) participated. Two patients did not attend CMR3, one due to discontinuation of GH replacement (patient 9) and one for personal reasons (patient 7). The mean age was 55 years (SD ± 13.4), mean duration of GHD was 6.1 years (SD ± 5). Six patients had previously undergone treatment for pituitary tumours, one had previous hypophysitis and two had undergone radiotherapy for meningioma. All patients had at least one other pituitary hormone deficiency; these were appropriately replaced at stable doses for at least four weeks before CMR1. GH therapy was commenced at a low dose and titrated, aiming for an IGF-1 between the median and upper limit of the age- and sex-adjusted reference ranges [23]. For patient characteristics see Table 1 *.*

**Table 1:** Characteristics of patients with GHD

Pt	Age	Sex	Symptom duration (years)	Reason for GHD	Other medical conditions	Medications	Visits
1	73	Male	3	NFPA, TSS 2005, EBRT 2005	Mild OSA	Levothyroxine	All
	—	—	—	—	—	Testosterone	—
	—	—	—	—	—	Aspirin	—
	—	—	—	—	—	Hydrocortisone	—
2	49	Male	18	Prolactinoma, medical therapy	Type 2 DM hypertension	Ramipril	All
	—	—	—	—	—	Candesartan	—
	—	—	—	—	—	Levothyroxine	—
	—	—	—	—	—	Aspirin	—
	—	—	—	—	—	Bromocriptine	—
	—	—	—	—	—	Metformin	—
3	56	Male	10	NFPA, TSS 1998, EBRT 1998		Levothyroxine	All
	—	—	—	—	—	Hydrocortisone	—
	—	—	—	—	—	Testosterone	—
	—	—	—	—	—	Aspirin	—
	—	—	—	—	—	Lansoprazole	—
4	51	Male	3	NFPA, TSS 2006	Hypothyroidism	Levothyroxine	All
	—	—	—	—	High cholesterol	Testosterone	—
	—	—	—	—	—	Simvastatin	—
5	50	Female	4	NFPA, craniotomy 1991, EBRT 1991, TSS 1992, TSS 2005, EBRT 2008	Renal stones	HRT	All
6	58	Male	2	NFPA, TSS 2003	High cholesterol	Levothyroxine	All
	—	—	—	—	—	Hydrocortisone	—
	—	—	—	—	—	Testosterone	—
	—	—	—	—	—	Atorvastatin	—
7	34	Female	6	Hypophysitis during pregnancy 2003	Depression	Levothyroxine	1, 2
	—	—	—	—	—	Seroxat	—
8	76	Male	3	NFPA, pituitary apoplexy 2007	—	Levothyroxine	All
	—	—	—	—	—	Hydrocortisone	—
	—	—	—	—	—	Testosterone	—
9	57	Male	9	Meningioma, EBRT 2000	Disordered sleep	Levothyroxine	1, 2
	—	—	—	—	—	Carbamazepine	—
	—	—	—	—	—	Amitripyline	—
10	37	Male	3	Meningioma, Surgery, Radiotherapy 2006	Acute lymphoblastic leukaemia-chemotherapy and cranial irradiation 1977	Levothyroxine	All
	—	—	—	—	—	Hydrocortisone	—
	—	—	—	—	—	Testosterone	—

*Pt* patient identification number, *Symptom duration* number of years experiencing symptoms of GHD, *NFPA* non-functioning pituitary adenoma, *TSS* transsphenoidal surgery, *EBRT* external beam radiotherapy, *OSA* obstructive sleep apnoea, *HRT* hormone replacement therapy with oestrogens, *visits* number of CMR visits attended

#### Control group

Ten individually age-matched controls and sex-matched controls were recruited. Controls underwent one non-contrast CMR study and provided serum for IGF-1 levels. Controls did not receive adenosine during CMR. For their characteristics see Table 2.

**Table 2:** Comparison of GHD patients and their controls

	Controls, *n* = 10	Patients, *n* = 10	*p* value
Gender (male/female)	8/2	8/2	NA
Age (years)	54 (12.1)	55 (13.4)	0.890
Male weight (kg)	71.2 (9.5)	87.8 (11.6)	**0.007**
Female weight (kg)	58.8 (8.1)	75.0 (19.8)	0.395
Male height (cm)	175.9 (4.6)	173.8 (9.6)	0.580
Female height (cm)	166.5 (4.9)	156.5 (2.1)	0.120
Male body mass index (kg/m^2^)	23.1 (3.5)	29.1 (3.5)	**0.004**
Female body mass index (kg/m^2^)	21.3 (4.2)	30.5 (7.3)	0.260
Systolic blood pressure (mm Hg)	133 (14)	135 (19)	0.863
Diastolic blood pressure (mm Hg)	82 (10)	83 (12)	0.811
Known hypertension (number)	0	1	NA
Known diabetes (number)	0	1	NA
Smoker yes/no/ex (number)	3/5/2	2/6/2	NA

Data are given as mean (SD)

### Cardiac magnetic resonance

CMR was performed at an experienced centre using a Philips Achieva CV 1.5 Tesla magnetic resonance imaging (MRI) scanner (Philips Medical Systems, Guildford, UK). Standard protocols were used to generate cine images. These were analysed using dedicated software (cmr42-Circle Cardiovascular Imaging Incorporated, Canada) to calculate the following cardiac parameters: LV-end diastolic volume (EDV), end systolic volume (ESV), ejection fraction (EF), stroke volume (SV), cardiac output, LV mass (M); RV-EDV, ESV, EF, SV. All except for EF were indexed (normalised) to body surface area (BSA) using the Mostellar formula and suffixed “i” [24]. CMR images were analysed by a single observer, blinded to the underlying diagnosis. To assess for bias and error in the measurement of LV mass and to ensure reliability, inter-observer variability was investigated by the independent analysis of 50 % of scans by an experienced colleague, blinded to the original measurements. There were no significant differences between these two sets of results on Bland–Altman analysis.

### Stress perfusion imaging

The GHD group underwent first pass perfusion imaging with a bolus of gadolinium contrast agent (0.05 mmol/kg), during an intravenous infusion of adenosine (140 µg/kg/min, given for 3 min). Three short axis slices were acquired per cardiac cycle using a steady-state prospectively gated turbo field echo sequence. Late gadolinium images were acquired at least 10 min following the injection of a further 0.15 mmol/kg of contrast agent, using an inversion recovery sequence. Perfusion and late gadolinium images were qualitatively assessed by an experienced cardiologist blinded to the underlying diagnosis.

### Aortic area

Aortic dimensions were obtained from MRI scout images. Internal distances in lateral and anterior–posterior planes were measured at the level of the pulmonary bifurcation. Area was calculated and indexed to BSA.

### IGF-1 measurements

IGF-1 levels were measured using a chemiluminescent immunoassay (Siemens, Surrey, UK), reporting IGF-1 levels between 25–1600 ng/ml. Age-adjusted reference and sex-adjusted reference ranges were used. IGF-1 standard deviation scores (SDS) were calculated using a standardised reference table from the assay manufacturer.

### Quality of life questionnaires

The QoL assessment of GH deficiency in adults (AGHDA) was used for patients with GHD, in which a lower result indicates a better QoL [25].

### Adverse outcomes

There were no significant adverse outcomes from this study. The only reported side effects were discomfort during venous cannulation and an uncomfortable sensation when receiving either the adenosine or the gadolinium contrast infusion. Effects from both were classed as mild.

### Statistics

Patients in whom new diagnoses of a non-GH related cardiac or aortic pathology were made during the study had their results excluded from the cardiac indices or aortic calculations, respectively. Details of any results excluded are given in the discussion. The Student’s *t*-test was used to compare patients with controls. Repeated measures analysis of variance (ANOVA) were used to compare repeated CMR studies. Paired *t*-tests were used to compare IGF-1 SDS and QoL scores at the beginning and end of the study. Pearson’s correlation test was used to compare IGF-1 SDS and left ventricular mass index (LVMi). Data are reported as mean and standard deviation, except where specified. Analysis was performed using StatsDirect (Ian Buchan, Cambridge, UK) and Prism (GraphPad, California, USA). A *p* value of <0.05 was deemed to be statistically significant.

## Results

### Baseline anthropometric comparison of patients and controls

Male GHD patients were heavier than their controls (*p* = 0.007) and had a greater body mass index (*p* = 0.004; Table 2). There were no other significant differences between the patients and their respective control groups.

### Baseline LV characteristics

**Fig. 1:**
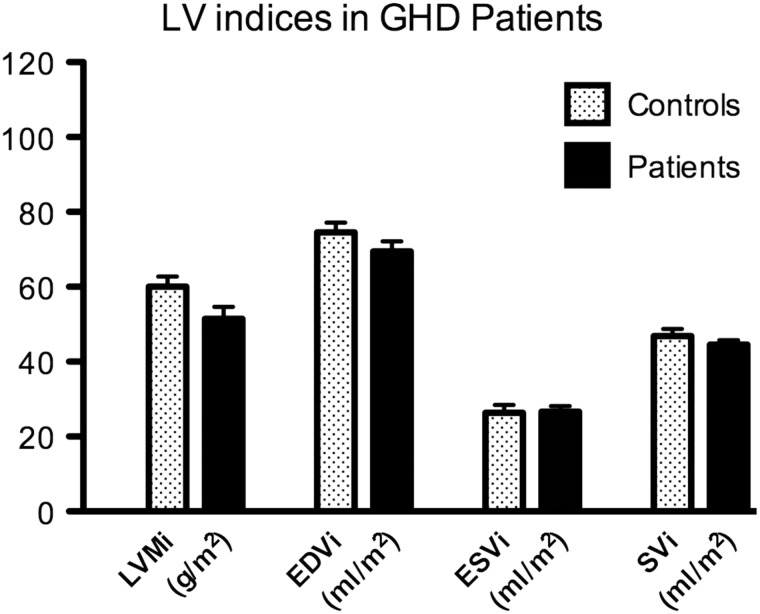
Baseline LV indices in GHD patients compared to controls. There is a strong trend towards reduced LVMi in GHD patients (*p* = 0.062). Data are presented as means, error bars represent SEM

GHD patients demonstrated a strong trend towards reduced LVMi (51.4 ± 3.2 vs. 60.0 ± 2.8 g/m^2^, *p* = 0.0615; Fig. 1)*.* No other differences in LV indices were seen: end-diastolic volume index (EDVi) 69.5 ± 2.7 vs. 74.5 ± 2.6 ml/m^2^, *p* = 0.194; ESVi 26.6 ± 1.6 vs. 26.4 ± 2.1 ml/m^2^, *p* = 0.9288; stroke volume index (SVi) 44.5 ± 1.2 vs. 46.9 ± 1.9 ml/m^2^, *p* = 0.305; cardiac index (CI) 2.96 ± 0.2 vs. 3.33 ± 0.2 ml/min/m^2^, *p* = 0.218; EF 64.6 ± 2.2 vs. 62.9 ± 0.9 %, *p* = 0.497. The control group had mean LVMi within the normal range [26].

### LV changes at 1 year

**Fig. 2:**
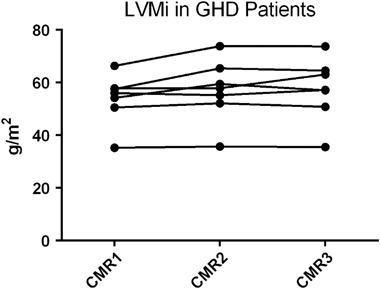
LVMi of GHD patients at baseline (CMR1), at 6 months of treatment (CMR2) and at 12 months of treatment (CMR3). There was an increase in LVMi with treatment compared to baseline (*p* = 0.0229)

Eight patients with GHD attended all three scans, of which one needed to be excluded (supplementary Table 1). In these seven patients, LVMi increased after 1 year of treatment (53.8 ± 9.5 vs. 57.0 ± 11.8 vs. 57.3 ± 12.1 g/m^2^, ANOVA *p* = 0.0229; Fig. 2). There were no changes in other parameters. The trend towards a reduced LVMi in GHD patients compared to controls at the beginning of the study, was lost at 1 year (57.3 vs. 59.9 g/m^2^, *p* = 0.666).

### Aortic area

**Fig. 3:**
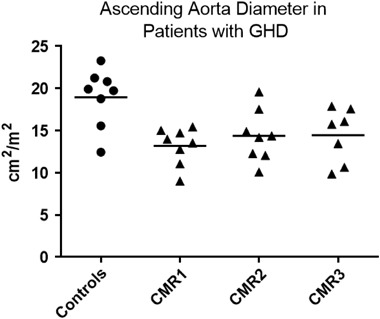
BSA-indexed ascending aorta area in patients with GHD. Patients have significantly lower aortic area than controls. There is no change in aortic area with 1 year of GH therapy. Line shows mean

Patients with GHD demonstrated reduced AAi compared to controls (13.2 ± 0.8 vs. 19.0 ± 1.2 cm^2^/m^2^, *p* = 0.001). There was no change in AAi with 1 year of GH therapy (*p* = 0.384; Fig. 3).

### Baseline RV characteristics

**Fig. 4:**
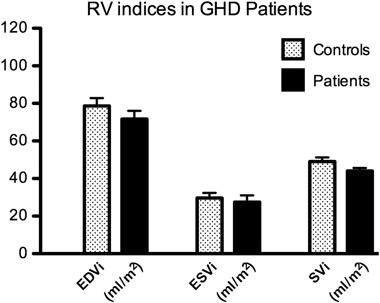
Baseline RV volume indices in patients with GHD. Patients with GHD show a trend towards reduced SVi (*p* = 0.0793). Data are presented as means, error bars represent SEM

GHD patients showed a trend towards reduced SVi (44.1 ± 1.5 vs. 49.1 ± 2.2 ml/m^2^, *p* = 0.079; Fig. 4). There were no differences in other parameters: EDVi 71.7 ± 4.4 vs. 78.6 ± 4.3 ml/m^2^, *p* = 0.275; ESVi 27.5 ± 3.6 vs. 29.7 ± 2.6 ml/m^2^, *p* = 0.629; EF 62.8 ± 2.6 vs. 63.0 ± 1.9 %, *p* = 0.946.

### RV changes at 1 year

Patients with GHD showed a trend towards increased RV-EDVi with GH replacement (CMR1 vs. CMR2 vs. CMR3: 70.3 ± 3.4 vs. 74.3 ± 3.1 vs. 73.8 ± 2.7 ml/m^2^, *p* = 0.062) but no increase in SVi (45.8 ± 1.1 vs. 46.9 ± 1.8 vs. 48.6 ± 1.27 ml/m^2^, *p* = 0.283). There were no differences in other parameters.

### IGF-1 changes with treatment

Patients with GHD demonstrated a significant increase in IGF-1 SDS with 1 year of GH treatment (*p* = 0.0032): baseline mean IGF-1 SDS − 1.60; 1 year + 0.61. Patient 3 was over-replaced (IGF-1 SDS 3.02) so the GH dose was reduced. Mean GH dose was 0.33 mg/day in males and 0.50 mg/day in females (Table 3).

**Table 3:** Treatment of patients with GHD

Pt	Sex	Final GH dose (mg/day)	Initial IGF1 SDS	Final IGF1 SDS
1	Male	0.30	−1.70	0.40
2	Male	0.30	−1.47	0.06
3	Male	0.50	−2.23	3.02
4	Male	0.35	−0.46	−0.01
5	Female	0.60	−3.58	−1.83
6	Male	0.20	−0.43	0.86
7	Female	0.40	−2.19	−1.46
8	Male	0.50	−2.39	−0.27
9	Male	0.20	0.41	1.44
10	Male	0.30	−1.97	1.88

Where patients dropped out before the end of the study, IGF1 level at visit 2 is given. IGF1 given as SDS

*Pt* patient identification number

### Late gadolinium enhancement

Two patients with GHD (Patients 1 and 3) demonstrated LGE. Patient 1 had extensive, transmural LGE, measuring 20.1 cm^3^. This matched an area of mildly reduced perfusion but was not associated with a wall motion abnormality. Patient 3 had mild, patchy, basal and mid-inferolateral LGE.

### Perfusion CMR

Three patients (patients 1, 3 and 8) with GHD had evidence of stress-induced myocardial perfusion defects. Patient 1 and 8 had mild abnormalities but were symptom-free, thus no further investigations were performed. Patient 3 demonstrated more marked perfusion abnormalities and underwent computed tomography (CT) angiography and clinical review by a cardiologist. This patient demonstrated a non-flow-limiting lesion on CT angiography.

### Quality of life

Patients with GHD demonstrated a significant improvement in AGHDA score with 1 year of GH treatment (baseline median AGHDA 17 vs. 1 year median AGHDA 5.5, *p* = 0.0025).

### Exclusions

Patients in whom a new, non-GH-related, cardiac diagnosis was made were excluded from relevant analyses: patient 9 had a bicuspid aortic valve and was excluded from AA analysis; patient 2 was discovered to have hypertension and diabetes and, therefore, was excluded from the cardiac mass and indices analysis. With patient 2 included within the calculations (Supplementary Table 1), LVMi in GHD patients is similar to controls (53.9 ± 12.0 vs. 59.5 ± 8.0 g/m^2^, *p* = 0.232). There is, however, a significant increase in LVMi in GHD patients after 6 months of treatment (53.9 ± 12.0 vs. 56.6 ± 12.5 g/m^2^, *p* = 0.0479), as well as 12 months of treatment when compared to their initial LVMi (56.6 ± 11.8 vs. 59.5 ± 12.8 g/m^2^, *p* = 0.0383). The other LV parameter calculations remained similar when compared to baseline.

## Discussion

The cardiac complications of GHD remain poorly understood [1, 15]. GHD affects many physiological systems and is associated with a range of risk factors that may increase the risk of ischaemic cardiac disease. CMR is established as the most accurate method for the assessment of cardiac mass and provides a wealth of further information on function, ischaemia and localised fibrosis, which would previously have required additional or invasive testing [27, 28]. Patients with GHD demonstrated a strong trend towards reduced LVMi. LVMi increased with 1 year of GH replacement. Abnormalities during stress CMR were seen in three patients and abnormalities on LGE imaging were seen in two patients.

Recruitment of treatment-naive patients with AO-GHD was particularly difficult. Since it has been shown that replacing GH in adults with GHD is beneficial, most patients fulfilling replacement criteria have commenced treatment, reducing patient availability for studies [29, 30]. Some patients with very low-baseline IGF-1 experienced significant clinical benefit from the treatment (as assessed by AGHDA scores) although IGF-1 did not reach the normal range. Furthermore, it should be noted that although all the patients met the criteria for severe GHD as per NICE (National Institute of Health and Clinical Excellence) guidelines, some patients did not have IGF-1 levels more than 2 standard deviations beneath the median, demonstrating the variability of IGF-1 production at different serum GH levels. It is unclear if this influenced some findings. However, despite small numbers of patients in this preliminary study, we were able to demonstrate a strong trend towards reduced LVMi in AO-GHD patients.

To date, it is well established that untreated CO-GHD reduces cardiac mass but whether this occurs in AO-GHD has not been clearly demonstrated [11]. Some echo studies in patients with GHD show increased LV mass with 1 year of GH replacement while other studies demonstrate no change [6–10]. However, these historical studies mainly describe patients receiving supraphysiological GH doses far in excess of current UK guidelines (as was past practice) and contain a mixture of both AO- and CO-GHD patients, which may confound results. Despite using lower GH doses than many older studies, we were able to show that GHD patients demonstrate a significant increase in LVMi with 1 year of GH treatment. Andreasson’s CMR study of 16 AO-GHD patients receiving similar doses of GH to this study showed a strong trend towards increased LV mass over 1 year (*p* = 0.059), validating this finding [22]. The improved accuracy of CMR over previous methods of measuring cardiac mass has been able to show that untreated AO-GHD is likely to be associated with reduced cardiac mass, although larger numbers would be needed to fully demonstrate this, and that GH replacement increases cardiac mass in GHD.

The mechanism behind reduced cardiac mass in GHD is likely to be multi-factorial. Withdrawal of GH replacement in teenagers with GHD reduces skeletal muscle fibre diameter [31]; whereas, high-dose GH replacement in patients with AO-GHD increases the area of both type I and type II skeletal muscle fibres [32]. In animal models of GHD, cardiomyocyte area is reduced and increases with GH therapy [33]. These findings suggest that the reduced LVMi seen in this study is due to reduced cardiac muscle fibre diameter, which increases with GH replacement.

Age may be an influential factor in the effects of GHD on LV mass. The mean age of patients in this study was 55 years (range 34–76). A study of older patients with GHD demonstrated no difference in LV mass compared to controls [34]; however, a study of younger patients showed reduced LV mass, although this contained both AO-GHD patients and CO-GHD patients [6]. Furthermore, GH replacement has been shown to increase LV mass in younger patients, when similar findings were not found in a study of older patients, although different surrogates of mass were measured [6, 10]. It is likely that achievement of peak cardiac mass remains under the influence of GH in young adulthood but that in older individuals, factors other than GH, such as blood pressure, have a more significant effect of cardiac mass than the effect of GHD.

### Right ventricular findings

CMR is considered the most accurate and reproducible method for assessing RV volume parameters [35–37]. Patients with GHD demonstrated reduced RV SVi. There are no published works examining RV volume parameters by CMR in patients with GHD. However, as GH excess is known to produce biventricular cardiac involvement, it seems reasonable that lack of GH would also produce the biventricular changes seen here [1, 15, 38].

One year of GH replacement in GHD did not result in significant changes in the RV parameters that were measured, except a trend towards increasing RV-EDVi. However, as RV measurements pose technical challenges and are more prone to error than those on the LV; larger studies may be required to ascertain if GH replacement increases RV cardiac indices [36].

### Late gadolinium enhancement

LGE utilises the gadolinium washout characteristics of collagen. Areas of fibrosis contain increased collagen and retain gadolinium-based contrast longer than non-affected areas [39, 40]. Cardiac fibrosis is not a reported feature of GHD. Of the two patients with GHD who demonstrated LGE, one had a missed previous myocardial infarct and one had mild, patchy fibrosis and a myocardial perfusion defect. Ischaemic heart disease was the probable cause of LGE in these two patients.

### Perfusion CMR

Cardiovascular mortality appears to be increased in hypopituitary patients taking glucocorticoids, sex-steroids and thyroxine replacement, and GH treatment may improve it to background rates [41–43]. This is thought to be due in part to an increase in risk factors for cardiac ischaemia, such as raised total cholesterol, low-density lipoprotein-cholesterol and apolipoprotein B in GHD [44, 45]. In our study, one patient with GHD had reversible ischaemia and one a previous myocardial infarction on LGE. CMR may be useful when assessing patients with GHD, providing information about underlying cardiovascular disease as well as identifying other cardiac problems, although at present there does not seem to be a role for such imaging in the routine care of the GHD patient. Given the potential for ischaemic heart disease in this population, a low threshold for 3-hydroxy-3-methylglutaryl-coenzymeA (HMG-CoA) reductase inhibitor therapy is indicated.

### Aortic area

CMR is recognised as an accurate method for assessing aortic dimensions and has been used to study the aorta in both Marfan and Turner syndromes [46, 47]. In this study, patients with GHD demonstrated significantly reduced ascending AAi. This is a novel finding. Reduced aortic diameter has been demonstrated in adults with CO-GHD, which may reflect impaired development [48]. The only study that looked at aortic diameter in patients with mainly AO-GHD did not report any increase with GH replacement but did not compare the aortic diameters of patients with GHD to those of healthy individuals [8]. In our study, GH replacement did not increase AA, confirming findings from another study that failed to show an increase, even with 5 years of GH therapy [8]. The mechanisms behind the reduction seen in AA in AO-GHD patients are not clear. Reduced AA could be due to an impact of GH on blood vessel development. Indeed, there are data to suggest that IGF-1 is involved in angiogenesis and that children with GHD have reduced retinal vascularisation [49]. However, our patients had an average pre-diagnosis phase of 6.1 years, which does not seem long enough to have this impact on AA. We cannot rule out that this might not be related to endocrine abnormalities, although unlikely. An alternative hypothesis for reduced AA is long-term reduced blood pressure but our patient group did not have a significantly different blood pressure compared to controls and there is no convincing evidence that AO-GHD is associated with a lower blood pressure.

### Exclusions

Patients in whom a new, non-GH-related, cardiac diagnosis was made were excluded from relevant analyses: patient 2 had LV hypertrophy thought to be due to hypertension and diabetes and therefore was excluded from the cardiac mass and indices analysis; patient 9 had a bicuspid aortic valve and was excluded from AA analysis.

With patient 2 included within the calculations for this study, the trend of reduced LVMi in GHD patients is lost (53.9 ± 12.0 vs. 59.5 ± 8.0 g/m^2^, *p* = 0.232).There is, however, a significant increase in LVMi in GHD patients after 6 months of treatment (53.9 ± 12.0 vs. 56.6 ± 12.5 g/m^2^, *p* = 0.0479) as well as 12 months of treatment when compared to their initial LVMi (56.6 ± 11.8 vs. 59.5 ± 12.8 g/m^2^, *p* = 0.0383). Over the 12 months, ANOVA calculations showed a trend towards increasing LVMi in GHD patients (56.6 ± 11.8 vs. 59.1 ± 12.4 vs. 59.5 ± 12.8 g/m^2^, ANOVA *p* = 0.063). There remained no significant difference in all other LV parameters when compared to baseline.

### Limitations

We understand that this study was limited by small patient numbers, a few exclusions and limited length of follow-up, and future studies are needed to confirm these data.

## Conclusions

We suggest that CMR could be used to identify and track the cardiac changes that occur in patients with GHD. The accurate assessment of LV mass by CMR allowed for the demonstration of a trend towards reduced cardiac mass in patients with AO-GHD and increase in cardiac mass with GH replacement. RV-EDV was reduced in GHD and reduced AAi was demonstrated in GHD patients.

## Supplementary material

Supplementary Table 1
