# Supplementary material for: Characterisation of myocardial structure and function in adult-onset growth hormone deficiency using cardiac magnetic resonance
Source: Endocrine. 2016 Aug 17;54(3):778–87. doi: 10.1007/s12020-016-1067-6 (PMC5107200; doi:10.1007/s12020-016-1067-6)
Supplement: Supplementary file 1 — Supplementary Table 1 [file 12020_2016_1067_MOESM1_ESM.docx]

Supplementary Table 1

LVMi parameters for GHD patients and controls

| **Patient** | **Scan 1** | **Scan 2** | **Scan 3** | **Control** |
| --- | --- | --- | --- | --- |
| Patient 1 | 66.26 | 73.67 | 73.66 | 70.88 |
| **Patient 2 (excluded)** | **75.97** | **73.73** | **75.10** | **55.14** |
| Patient 3 | 54.07 | 59.38 | 56.96 | 55.60 |
| Patient 4 | 55.89 | 55.07 | 57.12 | 66.28 |
| Patient 5 | 35.17 | 35.64 | 35.46 | 53.25 |
| Patient 6 | 50.44 | 52.00 | 50.68 | 50.84 |
| Patient 7 | 39.47 | 40.14 |  | 57.52 |
| Patient 8 | 57.40 | 65.30 | 64.46 | 50.42 |
| Patient 9 | 46.32 | 53.33 |  | 63.27 |
| Patient 10 | 57.68 | 57.77 | 62.95 | 71.99 |
